# Population-based analysis of breast cancer incidence and mortality: overall and age-specific temporal trends over 40-year period in Girona, Spain

**DOI:** 10.1007/s10549-025-07704-8

**Published:** 2025-04-25

**Authors:** Arantza Sanvisens, Anna Vidal-Vila, Montse Puigdemont, Gemma Viñas, Ariadna Roqué-Lloveras, Sonia Del Barco, Ferran Pérez-Bueno, Jan Trallero, Rafael Marcos-Gragera, Gemma Renart

**Affiliations:** 1 Unitat d’Epidemiologia i Registre del Càncer de Girona, Institut Català d’Oncologia, Pla Director d’Oncologia, c/ del Sol 15, 1era planta, 17004 Girona, Spain; 2 Descriptive Epidemiology, Genetic and Cancer Prevention Group, Institut d’Investigació Biomèdica de Girona Dr. Josep Trueta (IDIBGI-CERCA), Girona, Spain; 3 Institut de Recerca Contra la Leucèmia Josep Carreras, Girona, Spain; 4 Departament d’Oncologia Mèdica, Institut Català d’Oncologia, Hospital Universitari Dr. Josep Trueta, Girona, Spain; 5 Precision Oncology Group (OncoGIR-Pro), Institut d’Investigació Biomèdica de Girona Dr. Josep Trueta (IDIBGI-CERCA), Girona, Spain; 6 Departament d’Anatomia Patològica, Hospital Universitari Dr. Josep Trueta, Girona, Spain; 7 Grup de Recerca en Estadística, Econometria i Salut (GRECS), Universitat de Girona, Girona, Spain; 8 Departament de Ciències Mèdiques, Universitat de Girona, Girona, Spain; 9 CIBER of Epidemiology and Public Health (CIBERESP), Madrid, Spain

**Keywords:** Breast cancer, Incidence, Mortality, Epidemiology, Temporal trends

## Abstract

**Purpose:**

Breast cancer (BC) incidence and mortality in women have changed over time. This study aims to analyze population-level incidence and mortality trends over 40 years of observation.

**Methods:**

Population-based study of BC conducted by Girona Cancer Registry covering the period 1980–2019. Age-standardized incidence and mortality rates were calculated. Poisson change-point regression models were used to analyze trends, calculating the annual percentage change (APC).

**Results:**

A total of 12,283 diagnoses of invasive BC between 1980 and 2019. The overall age-standardized incidence rate was 109.9 (95% confidence intervals (CI) 104.4; 115.4) cases per 100,000 women-years. Trend analyses showed a statistically significant incidence increase of 4.2% per year from 1980 to 1994 (95%CI 3.3; 5.1), and a stabilization between 1994 and 2019, with an APC of 0.28% (95%CI − 0.04; 0.56). These trends were similar for the age groups 0–49 years and 50–69 years. In women over 69 years of age, an increase in incidence of 4.4% (95%CI 2.8; 6.0) per year was observed between 1980 and 1995 followed by a non-statistically significant decrease of − 0.35% (95%CI − 0.86; 0.15) between 1995 and 2019. The overall age-standardized mortality rate was 30.3 (95%CI 29.3; 31.3) cases per 100,000 women-years. Mortality rate trends showed a statistically significant decrease of − 1.87% (95%CI − 2.38; − 1.37) per year since 1992.

**Conclusion:**

There has been a stabilization in the incidence of BC and a gradual decline in BC mortality in women. The introduction of mammography in the mid-1990s, alongside early detection and treatment due to screening programs may play a significant role in the reduction of BC burden in women of all ages.

**Supplementary Information:**

The online version contains supplementary material available at 10.1007/s10549-025-07704-8.

## Introduction

Breast cancer (BC) is one of the most studied cancers at all levels since it is the most common tumor in women. It is estimated that, in 2022, more than 2.3-million women worldwide were diagnosed with BC, being also the leading cause of cancer death in women with nearly 665,000 deaths [1]. In Spain, recent data from the Spanish Network of Cancer Registries (REDECAN) estimate that 36,000 women will be diagnosed with BC in 2024 [2], with approximately 6500 deaths from this cancer.

Although mammography was introduced into clinical practice in the 1950s and has evolved over time [3], BC diagnosis underwent major advancements thanks to technological improvements in the 1990s, with higher quality and higher resolution images, as well as the advent of digital mammography [3, 4]. The availability of mammograms and their quality led to earlier diagnosis of this neoplasm at earlier stages. In United States, a shift in the incidence trend of this cancer was observed at the end of the 80 s due to screening programs that were introduced in the mid-1980s. [5]. In addition, the implementation of screening programs has also marked a turning point in the epidemiology of the disease [6, 7].

Screening programs for BC, as well as continued advances in treatment, have led to a significant reduction in mortality from this cancer over time [8, 9]. However, this improvement in mortality has been uneven, varying according to the socioeconomic level and healthcare system of each country [9]. In Europe, the reduction in mortality attributable to screening programs has been estimated to be between 0.7% and 5.0% per year [10].

Lower mortality rates have been observed in countries with higher levels of primary healthcare coverage and a greater number of public cancer centers. In addition, in countries with a continuous reduction in mortality, have reported an early detection rate (stage I or II) of more than 60% [9]. In fact, women aged 50 to 69 who are invited to mammography screening experience an average of 23% reduction in the risk of dying from BC; furthermore, those who were diagnosed within the screening program have an even greater risk reduction, estimated at about 40% [11].

Mammography in Catalonia, Spain, began in the 1980s but expanded in the 1990s. In 1980, only 10 mammography units were available, while there were 134 by 2000 [12].

In the province of Girona, where this study was conducted, the BC Prevention Program (Programa de Detecció Precoç del Càncer de Mama, PDPCM) was gradually implemented in hospitals from 1999 to 2002 [13], and the digital mammography was introduced in 2005 [13]. Currently, the age range for participation in the BC screening program is between 50 and 69 years. The most recent data available on the program (2022) showed a participation rate of 60.5% and a coverage rate, including women who reported having a mammogram outside the program, of 68.8% [14]. However, there are no population-level results describing incidence trends in Catalonia since the start of mammography use to date.

In this evolving context, the aim of this study is to analyze changes in BC incidence and mortality over time using data from a large population-based cancer registry in Girona, Spain. In addition, these trends will be analyzed according to the stage of the disease at diagnosis.

## Methods

This study included BC diagnoses in women collected by the population-based Girona Cancer Registry (GCR), Spain, from January 1980 to December 2019. The GCR covers the population of the province of Girona, located in northeastern Spain, with 808,672 inhabitants in 2023, according to the Statistical Institute of Catalonia.

The GCR is part of the Spanish Network of Cancer Registries (REDECAN), the European Network of Cancer Registries (ENCR) and the International Association for Cancer Registries (IACR). Cancer registration procedures and coding rules were applied following the standards of the International Agency for Research on Cancer (IARC), and ENCR recommendations.

Cases registered with topographic code C50 according to the International Classification of Diseases for Oncology, 3rd Edition (first revision) (ICD-O-3), were included in this study. Only cases of BC with in situ or invasive behavior were recorded. Cases with hematological histologies were excluded.

For each tumor, age at diagnosis, date and basis of diagnosis, histology, behavior and stage at diagnosis were available. The stage was determined using the TNM classification system for malignant tumors, with the relevant edition based on the year of diagnosis. Information on stage has been available since 2000 and was obtained from medical records.

Cases of BC deaths in women in the province of Girona were obtained from the Official Mortality Registry of Catalonia, Department of Health of the Generalitat of Catalonia for the same period (1980–2019). These cases were identified by codes 174 and C50 according to the International Classification of Diseases (ICD) Version 9 and Version 10, respectively, and were stratified by age at death.

### Statistical analysis

Descriptive statistics were reported as median and interquartile range [IQR] for quantitative variables and as absolute frequencies and percentages for qualitative variables. Age at diagnosis was stratified according to the screening program participation categories: < 50, 50–69, and > 69 years. The date of diagnosis was stratified into 5-year periods.

Crude, age-specific, and age-standardized incidence and mortality rates were calculated with 95% confidence interval (95%CI) and expressed per 100,000 women–year (w–y). Standardized incidence rates were calculated by the direct method using the 2013 European standard population. The population at risk used was obtained from the National Statistics Institute (INE) [15]. Poisson regression models were used to analyze trends in incidence and mortality, assess the presence of a change point, and estimate the annual percentage change (APC). Data analyses were performed using R software (version 4.3.2).

## Results

### Incidence

A total of 13,398 BC diagnoses were included between 1980 and 2019, of which 12,283 (91.7%) were invasive and 1115 (8.3%) were tumors in situ. Quality indicators for the GCR showed that 97.5% of BC cases included were microscopically verified and 0.9% of cases were reported to the registry based solely on death certificates.

Overall, the median age at diagnosis was 60 years [IQR:49–72], with 3428 cases (25.6%) in the 0–49 age group, 6000 cases (44.8%) in the 50–69 age group, and 3970 cases (29.6%) in the > 69 age group.

Regarding invasive tumors, the overall incidence crude rate (CR) and age-standardized incidence rate (ASIR_E_) were 102.03 (95%CI 97.03; 107.03) and 109.90 (95%CI 104.39; 115.42) cases per 100,000 w–y, respectively; these rates doubled between 2015 and 2019 compared to 1980–1987. The age-specific incidence rates for the groups differ considerably, being 39.86 cases per 100,000 w–y in the 0-49 age group and almost six time higher in the group aged > 69 years. Table 1 shows the overall and age-specific incidence rates for invasive tumors; data for in situ tumors are shown in Supplementary Table 1.

**Table 1:** Number of cases, age-specific rates, crude rates and 2013 European population-standardized rates of incidence of invasive breast tumors, 1980–2019, Girona, Spain

Incidence	Number of cases	Age-specific rates^1^			CR (95%CI)^1^	ASIR_E_ (95%CI)^1^
		< 50	50–69	> 69		
Overall 1980–2019	12,283	39.86	202.20	239.91	102.03 (97.03; 107.03)	109.90 (104.39; 115.42)
1980–1984	676	22.46	120.18	141.59	56.52 (52.27; 60.77)	67.85 (62.66; 73.04)
1985–1989	925	25.50	149.40	211.73	74.16 (69.38; 78.94)	88.02 (82.28; 93.76)
1990–1994	1165	38.67	172.68	219.48	89.52 (84.38; 94.66)	101.58 (95.70; 107.46)
1995–1999	1420	39.54	205.76	259.79	104.07 (98.66; 109.48)	113.72 (107.76; 119.68)
2000–2004	1715	41.68	240.72	273.04	116.57 (111.06; 122.08)	124.81 (118.85; 130.77)
2005–2009	1860	42.6	216.3	250.14	107.36 (102.48; 112.24)	114.86 (109.57; 120.15)
2010–2014	2126	48.17	218.33	253.42	114.72 (109.84; 119.60)	117.04 (111.98; 122.10)
2015–2019	2396	50.89	247.07	248.63	127.92 (122.80; 133.04)	124.42 (119.38; 129.46)
Mortality						ASMR_E_ (95%CI)^1^
Overall 1980–2019	3422	6.06	44.86	110.49	28.42 (27.47; 29.37)	30.31 (29.29; 31.33)
1980–1984	291	6.08	48.74	91.37	24.33 (21.53; 27.13)	30.64 (26.99; 34.29)
1985–1989	379	8.34	57.76	106.22	30.39 (27.33; 33.45)	36.84 (33.08; 40.60)
1990–1994	425	6.48	58.04	129.69	32.66 (29.56; 35.76)	37.67 (34.06; 41.28)
1995–1999	440	6.81	50.47	126.93	32.25 (29.23; 35.27)	34.98 (31.69; 38.27)
2000–2004	470	5.88	51.30	120.21	31.95 (29.07; 34.83)	33.25 (30.21; 36.29)
2005–2009	404	4.23	37.69	91.34	23.32 (21.05; 25.59)	24.40 (21.99; 26.81)
2010–2014	498	6.35	35.87	106.83	26.87 (24.52; 29.22)	26.26 (23.91; 28.61)
2015–2019	515	5.18	32.68	111.55	27.50 (25.13; 29.87)	24.79 (22.59; 26.99)

*ASIR*_*E*_ age-standardized incidence rate using 2013 European standard population, *ASMR*_*E*_ age-standardized mortality rate using 2013 European standard population, *CI* confidence interval, *CR* crude rate

^1^Expressed as cases per 100,000 women-year

**Fig. 1:**
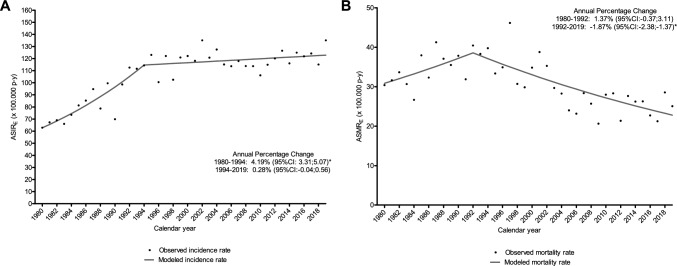
Overall incidence (**A**) and mortality (**B**) trends of invasive breast cancer, 1980–2019, Girona, Spain. *ASIR*_*E*_ age-standardized incidence rate using 2013 European standard population, *ASMR*_*E*_ age-standardized mortality rate using 2013 European standard population; *CI* confidence interval. *Statistically significant annual percentage change

**Fig. 2:**
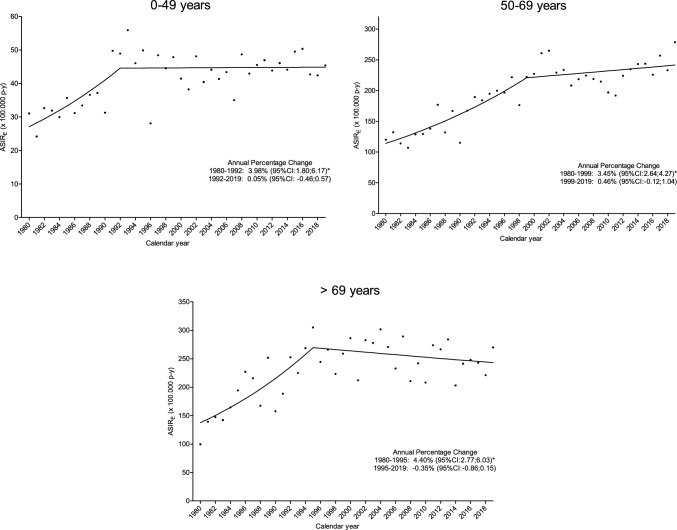
Incidence trends of invasive breast cancer according age groups, 1980–2019, Girona, Spain. *ASIR*_*E*_ age-standardized incidence rate using 2013 European standard population, *CI* confidence interval. *Statistically significant annual percentage change

Trend analyses of invasive BC showed a statistically significant increase in incidence of 4.19% per year from 1980 to 1994 (95%CI 3.31; 5.07), followed by stabilization between 1994 and 2019, with an APC of 0.28% (95%CI − 0.04; 0.56) (Fig. 1A). This trend pattern was similar for the all age groups, although the change point differed, occurring in 1992 for the 0–49 years group, in 1999 for 50–69 years and in 1995 for the > 69 years group. Incidence trends according to age group are shown in Fig. 2.

The ASIR_E_ according to stage varied significantly. For stage I the ASIR_E_ was 46.5 (95%CI 44.9; 48.2) cases per 100,000 w–y, similar to stage II, which was 42.5 (95%CI 41.0; 44.1) cases per 100,000 w–y. For stage III, the ASIR_E_ was 17.6 (95%CI 16.6; 18.6); for stage IV was 6.6 (95%CI 6.0; 7.3) and for stage 0 (in situ) was 14.7 (95%CI 13.7; 15.6) cases per 100,000 w–y. Trends by stage are shown in Supplementary Fig. 1. This figure shows that the major trend changes were observed in the early stages. Specifically, a statistically significant change in trend was observed in the diagnosis of in situ neoplasms, with an increase of 4.90% per year (95%CI 0.76; 9.02) from 2000 to 2009 and followed by a stabilization. For stage I, the APC for 2000–2019 was 1.54% per year (95%CI 0.91; 2.18). However, stage II showed a statistically significant annual decrease from 2000 to 2010 (APC: −2.16%, 95%CI −3.78; −0.54) and a subsequent non-statistically significant annual increase from 2010 onward (APC: 1.1%, 95%CI − 0.92; 3.13). Diagnoses in stages III and IV have remained stable over time (APC: − 0.18%, 95%CI − 1.17; 0.82 and APC: − 0.57%, 95%CI − 2.18; 1.04, respectively).

### Mortality

**Fig. 3:**
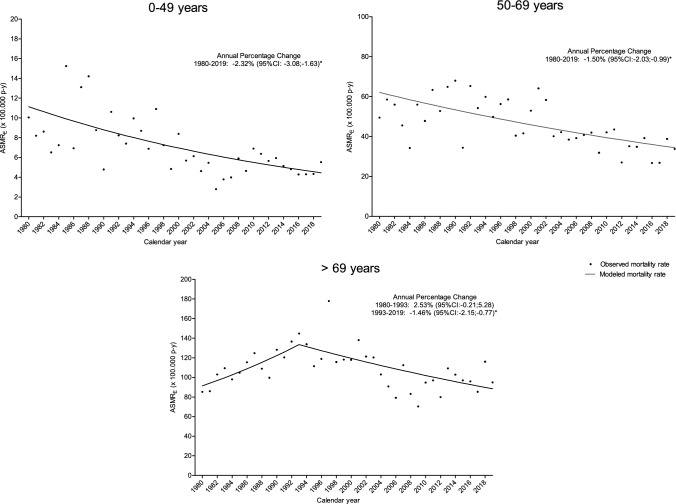
Mortality trends of breast cancer according age group, 1980–2019, Girona, Spain. *ASMR*_*E*_ age-standardized mortality rate using 2013 European standard population, *CI* confidence interval. *Statistically significant annual percentage change

A total of 3422 BC deaths were recorded between 1980 and 2019. The overall mortality CR and the age-standardized mortality rate (ASMR_E_) were 28.42 (95%CI 27.47; 29.37) and 30.31 (95%CI 29.29; 31.33) cases per 100,000 w–y, respectively (Table 1). Although incidence rates are similar for those aged 50–69 and over 69, mortality rates were more than double for the latter group. Trends in mortality rates showed a statistically significant decrease of −1.87% per year (95%CI −2.38; −1.37) per year since 1992 (Fig. 1B). According to age group the statistically significant decrease was observed overtime in the 0–49 age group (APC: − 2.32, 95%CI − 3.08; − 1.63), and in women aged 50–69 years (APC: − 1.50, 95%CI − 2.03; − 0.99), and since 1993 in those aged > 69 (APC: − 1.46, 95%CI − 2.15; − 0.77). Trends in mortality rates according to age group are shown in Fig. 3.

## Discussion

The results of this study describe the incidence and mortality of BC over 40 years using data from a consolidated Spanish population registry and mortality data from Catalonia. These findings depict the changes in the epidemiological indicators of BC disease over time, attributed to advances in molecular knowledge and improvements in diagnostic tools and treatment resources.

Overall, the incidence of invasive BC in Girona has increased over time, reaching 124 per 100,000 w–y in 2015–2019, a figure similar to those observed in other European regions [16, 17]. The data also indicate a change in incidence trend starting in 1994, with an annual increase of about 0.3% per year, primarily between women aged 50–69 and early-stage diagnoses. These results could be largely attributable to early detection and screening programs in Spain [18]. Specifically, in 50–69 age group, which corresponds to the age of participation in the screening program, a shift in trend was observed in 2000, coinciding with the implementation of the program in Girona.

The increase in incidence until the early 1990s is also observed in the other age groups; however, the incidence stabilizes thereafter. Several studies have attributed the increase in incidence to the growing prevalence of risk factors such as alcohol and tobacco use, obesity, or hormonal and reproductive factors [19, 20]. Nonetheless, few studies have described the subsequent stabilization; Leclere et al. [21], analyzed the trends in women younger than 40 years of age between 1990 and 2008 and reported a non-significant linear increase of 1.19% per year on average.

On the other hand, a comparison with studies from high-income countries, such as the United States and Western Europe, shows similar trends of stabilization and decline in incidence, indicating potential similarities in screening and public health interventions [22, 23].

Lima et al. [24], described a significant increase in incidence in Europe and Central Asia among women under 50, but stability when the data was adjusted for fertility rates: specifically for Spain, the results of Lima et al. for this age group are similar to those presented in our study. Nonetheless, the age distribution of BC diagnoses showed different patterns among countries [25]; in addition, the implementation and age of participation in screening programs also vary, limiting the comparability of incidence trend data [17, 26].

In a previous study with data from the same geographic area as this study examining in situ cancer cases between 1983 and 2007, a non-statistically significant APC of 3.6% from 1997 was observed. In the present analysis, which includes 40 years of observation, the incidence of stage 0 cases is stable since 1999 (APC = 0.6%). Furthermore, it is noteworthy that diagnoses in stages III and IV have remained stable over time, being consistent with other published series [27, 28]. Considering the significant increase in overall incidence over the years, this result supports that diagnosis in non-advanced stages has increased. In fact, it is estimated that organized screening with mammography reduces the proportion of diagnoses at advanced stages to the extent that at least 60% of invasive tumors are diagnosed at stage I or II [11].

In terms of mortality, the general downward trend in highly developed countries of Europe since the early 1990s has been extensively described, and is also reflected in our results and in the Spain as a whole; this decline is primarily due to early detection and advancements in treatment, including the introduction of trastuzumab at the early 2000s, among others [25, 29]. Specifically, by age groups, our study observes a gradual reduction in mortality rates throughout the analyzed period in both women under 50 years of age and in those between 50 and 69 years of age. However, for women over 69 years of age, the reduction has been observed since 1993; for this group, our results differ from those published by Lima et al. in 2021 [24], which reported an increase in mortality from this cancer in Europe between 1990 and 2017, although they combined data from Europe and Central Asia. Another study, which included data from 30 European countries between 1989 and 2006, showed that the results varied depending on the geographical area [29]. The observed decline in mortality in our study is consistent with findings from other high-income countries, where improvements in screening and treatment have similarly contributed to a decrease in mortality rates [30, 31]. The continued focus on early detection and advanced treatments, such as personalized medicine, is essential for maintaining these positive trends [29, 32].

Various published works confirm that young women who develop BC are more likely to die, primarily due to the biologic characteristics of the tumors that appear in this age group, which tend to have more aggressive phenotypes [19, 33]. However, when adjusted for tumor type, older women have the worst prognosis [34]. In our study, the mortality rate is approximately 6 cases per 100,000 w–y in women under 50 years of age and 110 cases per 100,000 w–y in women over 69 years of age, similar figures to those described in other European countries [35]. Additional studies should focus on age-specific risk factors and prognosis to develop targeted interventions that address the unique needs of both younger and older women [36, 37]. In addition, it is interesting to note that a previous study of our cohort showed that the long-term mortality in women with BC is increased mainly due to cardiovascular disease and the incidence of second tumors [38].

Nevertheless, BC prevention strategies that adopt a comprehensive approach including healthy lifestyle modifications, low-fat dietary recommendations, and new-generation of pharmacological options such as preventive endocrine therapy or oral conjugated equine estrogen are expected to significantly reduce mortality related to this disease [39, 40]. In fact, one of the pillars of the WHO Global BC Initiative focuses on health promotion and early detection through public education on risk reduction strategies (such as promoting breastfeeding, avoiding obesity, and limiting alcohol consumption) as a foundational step in cancer control [41].

One of the main limitations of this study is the lack of information on risk factors; in this context, the increase in incidence could reflect the increase in their prevalence. Furthermore, the data from the province of Girona are not representative of other regions of Spain due to the socio-demographic differences. However, this study provides population-level data based on a long observation period and serve as a reference for other regions with similar social and economic characteristics; in addition, it is valuable to provide useful information for health policy and services in Girona. While this study offers insightful population-level data, future research should integrate detailed risk factor analysis to provide a more comprehensive understanding of the trends observed [42].

In conclusion, this study demonstrates a positive evolution in the epidemiologic indicators of breast cancer in Girona, showing a stabilization in the incidence, a decrease in mortality and a significant improvement in early detection of the disease. Despite these relevant advances, there is still a need to develop preventive strategies aimed at promoting healthy lifestyles among the population, as well as tools to identify risk factors and poorer prognosis on a personalized basis.

## Supplementary Information

Below is the link to the electronic supplementary material.

Supplementary file1 (DOCX 48 kb)

## Data Availability

The datasets generated during and/or analyzed during the current study are not publicly available but are available from the corresponding author on reasonable request.
