# Supplementary material for: Population-based analysis of breast cancer incidence and mortality: overall and age-specific temporal trends over 40-year period in Girona, Spain
Source: Breast Cancer Res Treat. 2025 Apr 25;212(1):97–105. doi: 10.1007/s10549-025-07704-8 (PMC12086112; doi:10.1007/s10549-025-07704-8)
Supplement: Supplementary file 1 — Supplementary file1 (DOCX 48 kb) [file 10549_2025_7704_MOESM1_ESM.docx]

Supplementary Table 1. Number of cases, age-specific rates, crude rates and 2013 European population-adjusted rates of incidence of breast tumors in situ, 1980-2019, Girona, Spain

|  | Number of cases | Age-specific rates^1^ | | | CR (95%CI)^1^ | ASIR_E_ (95%CI)^1^ |
| --- | --- | --- | --- | --- | --- | --- |
|  |  | <50 | 50-59 | >69 |  |  |
| **Overall 1980-2019** | **1,115** | **4.18** | **25.24** | **9.03** | **9.45 (8.14,10.76)** | **10.33 (8.85, 11.81)** |
| 1981-1984 | 6 | 0.31 | 0.92 | 2.03 | 0.62 (0.13,1.11) | 0.88 (0.14, 1.62) |
| 1985-1989 | 20 | 1.21 | 1.78 | 3.59 | 1.60 (0.89,2.31) | 1.85 (1.03, 2.67) |
| 1990-1994 | 33 | 1.16 | 5.05 | 5.61 | 2.54 (1.68,3.40) | 2.91 (1.91, 3.91) |
| 1995-1999 | 132 | 4.36 | 26.82 | 9.14 | 9.67 (8.02,11.32) | 11.20 (9.28, 13.12) |
| 2000-2004 | 147 | 5.25 | 26.31 | 7.92 | 9.99 (8.38,11.60) | 11.07 (9.27, 12.87) |
| 2005-2009 | 244 | 7.32 | 36.85 | 12.34 | 14.08 (12.32,15.84) | 15.49 (13.53,17.45) |
| 2010-2014 | 273 | 4.83 | 43.81 | 13.26 | 14.73 (12.99,16.47) | 15.67 (13.79, 17.55) |
| 2015-2019 | 260 | 5.97 | 35.73 | 10.21 | 13.88 (12.19,15.57) | 13.75 (12.06, 15.44) |

^1^ expressed per 100,000 women-year

ASIR_E_: age-standardized incidence rate using 2013 European standard population; CI: confidence interval; CR: crude rate

Supplementary Figure 1. Incidence trends of breast cancer according to stage at diagnostic, 2000-2019, Girona, Spain

ASIR_E_: age-standardized incidence rate using 2013 European standard population; N: number of cases
